# Dematiaceous Fungal Infections in Hematopoietic Stem Cell Transplantation and Predisposing Hematological Conditions: A Systematic Review

**DOI:** 10.3390/jof12090689

**Published:** 2026-09-13

**Authors:** Christopher V. Radcliffe, Marwan M. Azar, Matthew Grant

**Affiliations:** 1 Section of Infectious Diseases, Department of Internal Medicine, Yale School of Medicine, Yale University, New Haven, CT 06510, USA; marwan.azar@yale.edu (M.M.A.); m.grant@yale.edu (M.G.); 2 GlaxoSmithKline, U.S. Medical Affairs, Durham, NC 27701, USA

**Keywords:** hematopoietic stem cell transplantation, phaeohyphomycosis, dematiaceous mold, invasive fungal infection, leukemia

## Abstract

Background: Dematiaceous fungi can cause invasive disease in patients with hematological malignancies and hematopoietic stem cell transplant (HSCT) recipients. Methods: To characterize the spectrum of disease in these populations, we performed a systematic review of PubMed and Scopus databases (searched 16 August 2026) and included reports with individualized data on dematiaceous fungal infections in adult HSCT recipients and patients with predisposing hematological conditions. A four-domain bias assessment was applied to reports, and data were summarized with descriptive statistics. Results: We identified 74 reports on 88 patients. The median age was 58 years, 61% were male, and 35% (31/88) were HSCT recipients. The most common genus was *Exophiala* (18/88; 20%). For 30 patients with information available, 25/30 (83%) received antifungal prophylaxis, and 64% (16/25) broke through mold-active prophylaxis. The most common clinical syndrome was mucocutaneous infection (40/88; 45%), followed by pulmonary (12/88; 14%) and sinusitis (11/88; 13%). Systemic antifungal therapy was administered in 92% (81/88) of infections. For 75 patients with information available, 63% were deceased at the time of reporting. Conclusions: Although limited by biases inherent to case reports, this review identified high all-cause mortality and frequent breakthrough infections. No funding sources were used, and this protocol was not registered.

## 1. Introduction

Dematiaceous fungi are generally defined by the production of melanin in both reproductive (ex: conidia) and non-reproductive structures (ex: hyphae) [1]. Fungal melanin serves as a virulence factor by resisting host phagocytes’ oxidative burst, and it is also theorized to protect against environmental stressors including heat and ultraviolet radiation [2,3]. The heterogeneous genera referred to as dematiaceous are often found as saprophytes in the environment, and common genera include *Exophiala*, *Alternaria*, and *Curvularia* [1]. Several members are globally distributed, but there is geographic variability for select species. For example, *Rhinocladiella mackenziei* is frequently reported in the Middle East, *Lomentospora prolificans* is more commonly reported in Australia, and certain members of the genus *Fonsecaea* are predominantly found in Central and South America [1,4,5,6]. Humans generally acquire infection via inhalation of aerosolized spores or from direct inoculation by environmental material (e.g., wood or soil) [7]. Rarely, nosocomial exposures cause dematiaceous fungal disease, and outbreaks have been reported in association with deionized water (*Exophiala jeanselmei*) and methylprednisolone injections (*Exserohilum rostratum*) [8,9].

Several clinical syndromes including skin and soft tissue infections (SSTIs), central nervous system (CNS) infections, endocarditis, sinusitis, pulmonary infections, and osteomyelitis have been caused by dematiaceous fungi [1,10,11,12,13]. Disseminated and CNS infections are associated with high mortality [11,12]. The net state of immunosuppression and the inciting fungal species both impact disease manifestations. Disseminated disease is more often reported in patients with immunocompromising conditions, but severe infections have been reported in patients with presumed immunocompetence [11]. Recent observations suggest that selective immunodeficiencies related to proteins and receptors involved in fungus-sensing signaling cascades (e.g., Dectin-1 and CARD9) can be implicated in severe disease manifestations [14,15]. The specific fungal species’ virulence and tissue tropism are also important determinants of the clinical syndrome and disease severity. For example, *Cladophialophora bantiana* is well known to be neurotropic and cause brain abscesses [16]. This may potentially stem from a different repertoire of enzymes that allow for neuroinvasion [17]. In contrast, *L. prolificans* commonly leads to disseminated disease, and this has been attributed to angioinvasion and host factors [11,18].

While the true incidence of dematiaceous fungal infections is not well characterized, a recent study using a United States national database identified hundreds of hospitalizations associated with phaeohyphomycosis and chromoblastomycosis between 2016 and 2021 [19]. Another updated multicenter retrospective study in Australia and New Zealand found roughly one-third of single-species non-*Aspergillus* mold infections to be caused by dematiaceous fungi [6]. Both studies included hematopoietic stem cell transplant (HSCT) recipients and other subpopulations at risk for invasive fungal disease [6,19]. Given the rising prevalence of immunocompromising conditions in the general population combined with potentially accelerated geographic spread of fungi driven by global warming, the importance of dematiaceous fungal infections is likely to increase over time [20,21]. Natural disasters including hurricanes, floods and tsunamis have been linked to clusters of dematiaceous fungal infections, and some data suggest seasonal variation in the titer of dematiaceous fungi in the environment [21,22].

The abovementioned factors underscore the importance of dematiaceous fungal infections. Clinical management of these infections may involve diagnosis, antifungal therapy, operative debridement, and reconstitution of host immunity. Supportive tissue histopathology with a species-level identification is ideal, and molecular methods combined with fungal morphology are often used for definitive identification [1]. Regarding non-invasive tests, the 1,3-beta-D-glucan (1,3-BDG) assay has some supportive data and demonstrated high sensitivity during an *E. rostratum* fungal meningitis outbreak [23], but no cross-reactivity with galactomannan was observed in vitro in a small case series on dematiaceous fungal infections [24]. After identification, dematiaceous genera remain challenging to treat due to genus- and species-level variation in antifungal susceptibility, and there are insufficient clinical outcomes data to support the determination of clinical breakpoints for antifungal agents’ minimum inhibitory concentrations (MICs) [25]. As a result, isolates cannot be labeled as susceptible, intermediate, or resistant to antifungal agents. Unlike clinical breakpoints, epidemiological cut-off values (ECVs) aim to define the wild-type MIC distribution and are entirely dependent on in vitro data that assess for phenotypic evidence of an acquired resistance mechanism [26]. For dematiaceous fungi, Clinical and Laboratory Standards Institute (CLSI) ECVs have only been determined for two members of the genus *Fonsecaea*, and none of the remaining genera have either clinical breakpoints or ECVs [27,28]. Triazoles, echinocandins, and amphotericin B are most commonly used, but principles of antifungal therapy are largely guided by expert opinion since robust evidence is lacking [25,29].

HSCT recipients and patients with predisposing hematological conditions are at high risk for several types of invasive fungal infections [30,31,32], and fungal disease contributes to post-transplant mortality [33,34]. These populations are subject to prolonged neutropenia and other forms of immune modulation which place them at higher risk for tissue-invasive fungal disease. Fungal infections in the pre- and post-transplant setting are important for a variety of malignant and non-malignant hematological conditions which may require transplantation or advanced immune effector cell therapies [35]. Transplant candidacy is contingent upon control and stabilization of infectious complications, so optimization of management is essential. At present, the guidelines for non-*Aspergillus* mold infections in HSCT recipients highlight challenges associated with selecting the optimal antifungal therapy for dematiaceous fungi [36], and triazole therapy in this population requires navigation of significant drug–drug interactions. Despite the high consequence of fungal disease in this population, data are limited for dematiaceous molds. 

To better understand the spectrum of dematiaceous fungal disease in one of the highest-risk subpopulations of immunocompromised patients, we performed a systematic review of cases in HSCT recipients and patients with predisposing hematological conditions. To our knowledge, this topic has not been recently reviewed. We aimed to describe the clinical syndromes, antifungal susceptibility data, management approaches, and outcomes.

## 2. Materials and Methods

### 2.1. Literature Search, Inclusion Criteria, and Bias Assessment

To identify eligible patients, we searched PubMed and Scopus databases with the following terms: ((“stem cell transplant*” OR “stem cell recipient” OR “stem cell” OR “allogeneic transplant*” OR “autologous transplant*” OR “syngeneic transplant*” OR “hematopoietic recipient” OR “hematopoietic transplant*” OR myeloproliferative OR myeloma OR “cord blood transplant*” OR “cord blood recipient” OR “myelodysplastic” OR “CAR-T” OR “CAR T-cell” OR “chimeric antigen receptor” OR leukemia OR leukemic OR lymphoma OR “aplastic anemia” OR myelofibrosis OR “chronic neutropenia” OR “prolonged neutropenia” OR neutropenia) AND (pigmented OR melanin OR melanized OR dark OR black OR brown OR dematiaceous OR “non-hyaline” OR phaeohyphomycosis OR pheohyphomycosis OR chromoblastomycosis)) AND (“mold” OR “molds” OR “fungus” OR “fungal” OR “fungi” OR “hyphae” OR “hyphal” OR “fungal elements” OR “fungal element”). The initial results were manually screened by one author (CVR) without the assistance of artificial intelligence or automation tools. To ensure inclusion of all relevant results, a second author (MG) reviewed search results which were excluded from the initial screen. Discrepant assessments were discussed between two authors (CVR and MG), and consensus was achieved. All years of publication were eligible for inclusion, and the database searches were both conducted on 16 August 2026. Our review protocol was not registered beforehand, and this review conformed to applicable sections of the 2020 Preferred Reporting Items for Systematic Review and Meta-Analysis statement (See Table A1) [37,38].

All English-language reports were eligible for inclusion, but only reports with individualized data on adult (age ≥ 18 years) patients who underwent HSCT and/or had an underlying hematological condition were included. Patients with hematological conditions were considered eligible for inclusion if the report used language compatible with active disease and/or the patient received disease-directed therapy during the 12 months preceding the diagnosis of dematiaceous fungal infection. Reports on patients with remote history of hematological conditions in remission without active therapy were excluded. The following classes of conditions were considered eligible for inclusion: leukemia, lymphoma, myelodysplastic syndrome, myeloproliferative neoplasm, aplastic anemia, and multiple myeloma. To be included, the report had to have a genus-level identification of the inciting fungal species. We recorded whether reports used matrix-assisted laser desorption/ionization-time of flight mass spectrometry (MALDI-TOF MS) and/or nucleic acid sequencing methods. Histopathology of melanized hyphae without further characterization was insufficient for inclusion. For the purposes of this review, only fungal genera with melanin production in both asexual and sexual structures were included as dematiaceous, and the production of melanin was insufficient for inclusion. This definition was chosen as some fungi produce melanin without macroscopic evidence of pigmentation on the top and reverse sides of colonies grown on mycological media. Furthermore, patients were required to have clinical evidence of tissue-invasive disease and include information on treatment(s) used. 

To assess the bias of included case reports and case series, we adapted a previously published bias assessment and focused on four domains: exposure, outcome, ability to draw inference for clinical practice, and length of follow-up [39]. For the exposure criterion to be fulfilled, authors had to report a probable exposure to the dematiaceous fungus (e.g., decaying wood) and/or explicitly report having inquired into one. For the criterion concerning ability to draw inference for clinical practice, reports had to explicitly name an antifungal agent used and/or whether procedural intervention occurred. For the outcome and length of follow-up criteria to be fulfilled, ≥6 months of follow-up after diagnosis and/or explicit report of death within this period had to occur. The bias assessment was developed by two authors (CVR and MG) and applied by one author (CVR) to each report. We also applied GRADE guidelines to assess the quality of reports, and this assessment was performed by two authors (CVR and MG) [40].

### 2.2. Data Collection and Classification

For each patient with individualized data available, we recorded age, gender, underlying hematological condition, presence of neutropenia (defined as absolute neutrophil count < 500/μL), chemotherapy, antifungal prophylaxis, and suspected exposures to dematiaceous fungi. When available, 1,3-BDG and galactomannan testing were recorded. For HSCT recipients, we recorded the type of transplantation, immunosuppression, time since transplantation, presence of graft-versus-host disease (GVHD) requiring immunosuppression, and evidence of disease relapse. Cases were classified as probable or proven according to 2020 consensus definitions offered by the European Organization for Research and Treatment of Cancer (EORTC) and the Mycoses Study Group Education and Research Consortium (MSGERC) [41]. If insufficient data were available to distinguish proven from probable, then cases were classified as possible. For patients receiving antifungal prophylaxis, breakthrough mold infection was defined as diagnosis of invasive fungal disease despite receiving one or more mold-active antifungal agents. We considered the following agents to have offered prophylactic mold coverage: amphotericin B, echinocandins, isavuconazole, posaconazole, and voriconazole. 

The names of fungal isolates are reported according to updated taxonomic classification from the National Library of Medicine National Center for Biotechnology Information database. When available, we recorded MIC values for antifungal susceptibility testing data as well as the exact wording of the reported method for MIC determination. No CLSI or European Committee of Antimicrobial Susceptibility Testing (EUCAST) clinical breakpoints are available for dematiaceous fungi, so MIC values were not interpreted as susceptible, intermediate, or resistant. The reports’ clinical syndromes were classified as mucocutaneous, sinusitis, fungemia, pulmonary, CNS, disseminated, and miscellaneous. The mucocutaneous class of infection encompassed SSTI as well as infections affecting mucosal surfaces (e.g., oropharyngeal ulcer). Disseminated infection was defined as an infection involving two or more non-contiguous organ systems, and the presence of positive blood cultures was insufficient to be classified as disseminated. Cases requiring adjudication were discussed between two authors (CVR and MG). When available, data on antifungal therapy and duration were recorded, as were the details of procedural interventions. The outcome of death at the time of reporting was recorded, as was the duration of follow-up. Mortality attributable to infection was not recorded due to anticipated heterogeneity of information available in reported cases.

### 2.3. Data Synthesis

Reports with individualized patient data meeting inclusion criteria were included in the data synthesis, and these reports were presented in a tabulated format. Descriptive statistics were performed for continuous variables using Microsoft Excel (version 16.105.1). Data imputation was not performed for missing variables. The sample size and large proportion of missing data precluded meta-analysis. 

## 3. Results

### 3.1. Search Results and Patient Population

**Figure 1:**
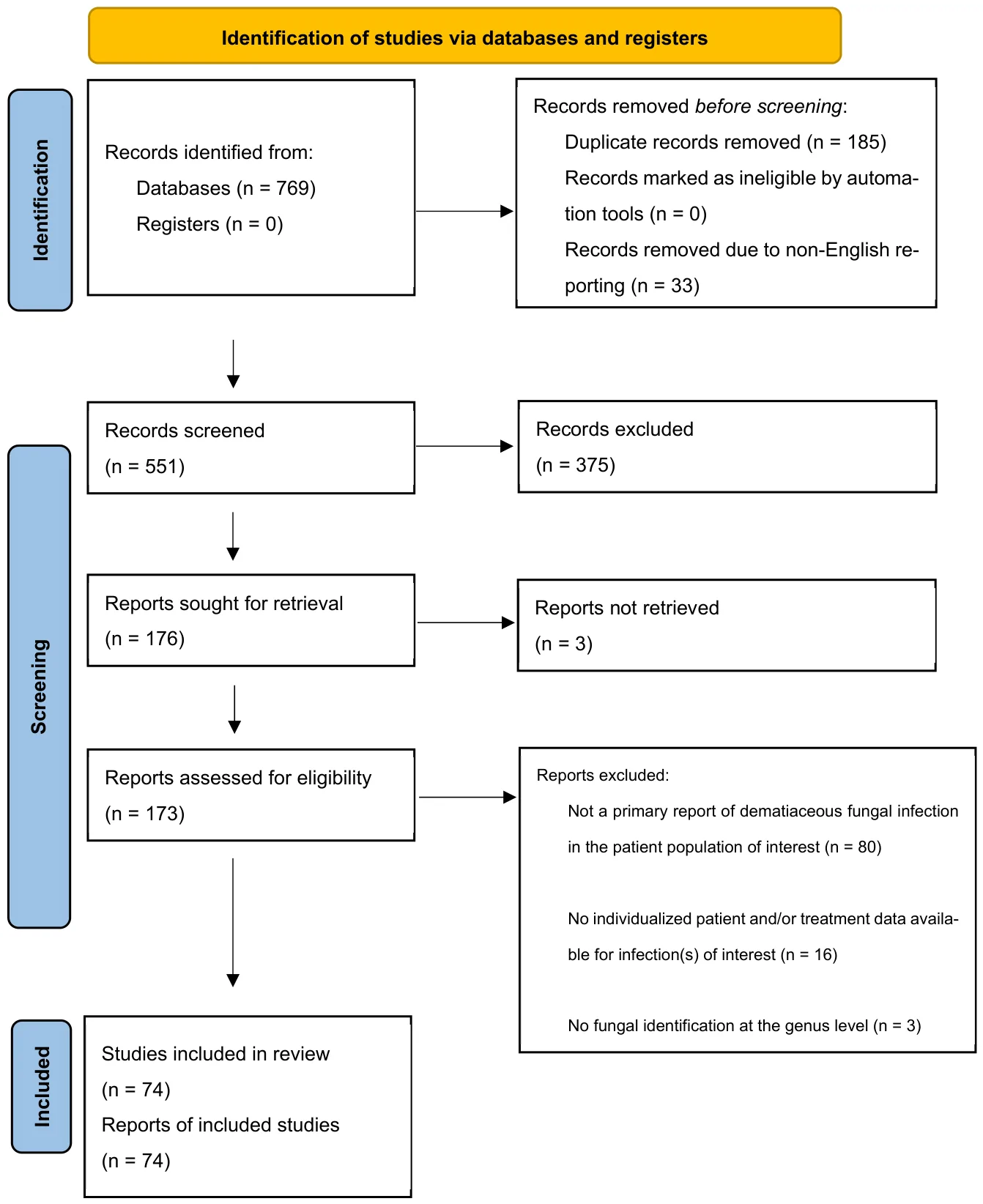
2020 Reporting Items for Systematic Reviews and Meta-Analyses diagram for inclusion of published reports on adult cases of dematiaceous fungal infections with individualized data in prespecified population of interest.

Our PubMed and Scopus search generated 769 reports published between 1950 and 2026. A total of 551 unique results were screened, and seventy-four reports met our prespecified inclusion criteria (Figure 1; Table A2) [24,42,43,44,45,46,47,48,49,50,51,52,53,54,55,56,57,58,59,60,61,62,63,64,65,66,67,68,69,70,71,72,73,74,75,76,77,78,79,80,81,82,83,84,85,86,87,88,89,90,91,92,93,94,95,96,97,98,99,100,101,102,103,104,105,106,107,108,109,110,111,112,113,114].

In total, three reports were excluded as no genus-level identifications were clearly specified for pigmented fungal isolates, and sixteen reports had no individualized patient data that fit our inclusion criteria [6,19,32,115,116,117,118,119,120,121,122,123,124,125,126,127,128,129,130]. All included reports were case reports, case series, or retrospective studies, and all reports had very low quality of evidence according to the GRADE assessment [40]. The resultant certainty in this body of evidence is very low. Using the modified bias assessment, 51 of 74 (69%) reports fulfilled ≥ 3 criteria. The 74 reports included information on a total of 88 patients with dematiaceous fungal infections (Table 1).

For the 84 of 88 patients with age reported, the median age was 58 years (range 18–84 years), and the majority were male (54/88; 61%). HSCT recipients accounted for 35% of cases (31/88). For non-HSCT recipients, the most common underlying condition was leukemia (22/57; 39%). For HSCT recipients with information available on the type of transplantation (22 of 31), the majority (18/22; 82%) were allogeneic stem cell transplant recipients. The median time between transplantation and diagnosis of dematiaceous fungal infection was two years for the 24 patients with information available, and 23% (7/31) had relapsed disease after transplantation. GVHD requiring immunosuppression was reported for 10 of 13 patients (77%) with information available. Immunosuppression regimens were reported for 39% of HSCT recipients (12/31), with glucocorticoids (10/12; 83%), calcineurin inhibitors (5/12; 42%), and mycophenolate (4/12; 33%) being common. Two of four patients with aplastic anemia had information on immunosuppression available, and both received cyclosporine with anti-thymocyte globulin during their infections. For non-HSCT recipients, chemotherapy was infrequently specified (33/53; 62%), and most (23/33; 70%) received cytotoxic chemotherapy. Only two of 33 (6%) received Bruton tyrosine kinase inhibitors. For the 30 patients with information on antifungal prophylaxis available, 25 of 30 (83%) received antifungal prophylaxis, and the most frequently used antifungal agents were fluconazole (8/30; 27%), posaconazole (5/30; 17%), voriconazole (5/30; 17%), and isavuconazole (3/30; 10%). 

### 3.2. Mycology and Susceptibility Data

Twenty-eight genera of dematiaceous fungi were represented in the 88 infections. Sequencing-based methods were used for fungal identification in 38 of 88 (43%). MALDI-TOF MS was used in 15% (13/88); however, one case reported that this method was unsuccessful in identifying *Parathyridaria percutanea*, so sequencing was required [88]. Both sequencing and MALDI-TOF MS were used in 10% (9/88) of cases. The commonly isolated genera were *Exophiala* (n = 18), *Microascus* (n = 9), *Alternaria* (n = 9), *Curvularia* (n = 8), *Exserohilum* (n = 6), and *Verruconis* (n = 5). There was one patient with co-infection due to *Alternaria* spp. and *Curvularia* spp. [84]. Two patients with SSTI each had two different species of dematiaceous fungi identified (*Phaeoacremonium parasiticum* and *Fonsecaea pedrosoi*; *Cladophialophora* spp. and *E. jeanselmei*) [49,107]. Ten patients (11%) were reported to have *Aspergillus* coinfections based on culture data, histopathology, and/or galactomannan testing [24,42,43,80,87,98].

Only 14 of 88 (16%) patients had information on 1,3-BDG testing reported, and nine of 14 (64%) had an elevated 1,3-BDG. The most common species reported in association with an elevated 1,3-BDG was *Exophiala dermatitidis* (n = 3) [72,74,75]. Regarding galactomannan testing, 16 of 88 (18%) patients had serum or bronchoalveolar lavage galactomannan information reported, and five of 16 (31%) reported an elevated value; however, four of five patients in this group were suspected to have concurrent aspergillosis [24,87,98].

Antifungal susceptibility testing results with MIC values were available in 35 reports, and 45 unique fungal isolates had MIC values reported. The most common standards organization referenced for MIC determination was CLSI (26/45; 58%). Table 2 summarizes antifungal susceptibility testing data for 45 isolates. At the time of writing, there are no CLSI or EUCAST clinical breakpoints available for the interpretation of MIC data obtained from dematiaceous fungal isolates, and none of the fungal isolates included in Table 2 have had ECVs determined.

### 3.3. Clinical Syndromes, Management, and Outcomes

Sixty-nine of 88 patients (78%) had proven or probable invasive fungal disease based on EORTC/MSGERC definitions. For the 25 patients reported to be on antifungal prophylaxis, 64% (16/25) represented breakthrough mold infections. Regarding exposures, the authors for 59% (52/88) of patients explicitly reported inquiring into potential sources for dematiaceous fungi. Half of the patients (25/52; 48%) had an identified exposure, and the most common exposures (14/25; 56%) were related to occupations and hobbies involving soil and/or plant matter. Only one HSCT recipient was described as an avid gardener [70]. Three SSTIs were associated with nosocomial exposures as the involved areas of skin were at a venipuncture site (n = 2) or a bone marrow biopsy site (n = 1) [47,52,90]. The most common clinical syndrome was mucocutaneous infection (40/88; 45%), with 39 of 40 (98%) representing SSTI. *Exophiala* (n = 10), *Alternaria* (n = 8), and *Colletotrichum* (n = 4) were most frequently isolated in the context of SSTI. The one mucosal surface infection was a tongue ulceration due to *Myrmecridium schulzeri* [43].

Pulmonary infections were the second-most common clinical syndrome (12/88; 14%), and 82% of infections (9/11) reported in HSCT recipients were accounted for by one retrospective study that reported nine *Microascus* infections from a single center in China [42]. The authors assessed for a nosocomial source and did not identify a common environmental exposure. For non-pulmonary infections without systemic dissemination, possible pulmonary involvement was reported in 12% (8/67) of patients without microbiological confirmation. Aside from mucocutaneous and pulmonary infections, sinusitis (11/88; 13%), disseminated infections (9/88; 10%), fungemia (6/88; 7%), and CNS infections (4/88; 5%) were also identified. Disseminated infections were caused by *L. prolificans* (n = 4), *E. dermatitidis* (n = 2), *Verruconis gallopava* (n = 2), and *Thermothelomyces thermophilus* (n = 1) [50,56,60,62,63,74,75,80,113]. The four CNS infections were caused by *C. bantiana* (n = 2), *Curvularia hawaiiensis* (n = 1), and *Rhinocladiella mackenziei* (n = 1) [53,64,110,112]. It is interesting to note that the patient with a *R. mackenziei* brain abscess also sustained a corneal ulceration from *Alternaria* spp. [53]. The miscellaneous class of infections included: lymphadenitis (n = 3) [54,55,91], isolated hepatic lesions (n = 1) [46], endophthalmitis (n = 1) [96], and tracheitis (n = 1) [51].

Systemic antifungal therapy was reportedly administered to 81 of 88 patients (92%). For the seven patients who did not receive antifungal therapy, three were diagnosed at autopsy, three underwent procedures for the involved skin and soft tissues, and one received intralesional injections of amphotericin B [49,50,97,101,105,112,113]. For the 80 patients with information on antifungal agents used as systemic treatment, 47 of 80 (59%) received two or more agents used in series and/or as combination therapy. Azoles were used in 79% (63/80) of infections, and 56% (45/80) received a systemic formulation of amphotericin B. Voriconazole (27/63; 43%) was the most common azole used, followed by posaconazole (17/63; 27%) and itraconazole (16/63; 25%). Only 23 of 81 (28%) patients had a defined duration of therapy reported, and the median duration was 1.2 months (range 0.2–7.3 months). In total, authors for 48 of 88 (55%) patients discussed whether surgical and procedural interventions were considered, and 37 of 88 (42%) patients underwent surgical and/or procedural interventions. Two of four CNS infections underwent neurosurgical intervention, as one was diagnosed at autopsy and the other had brainstem involvement not amenable to resection. In total, 82% (9/11) of sinusitis infections underwent nasal endoscopic interventions, and 18 of 39 (46%) SSTIs underwent excision, debridement, and/or drainage. Only two patients with pulmonary infections underwent surgical interventions [65,92]. The median duration of follow-up was 3 months (range 0.4–44.4 months) for the 35 patients with information available, and only 75 of 88 (85%) patients were explicitly reported as alive or dead at the time of reporting. In total, 47 of 75 (63%) patients were deceased at the time of reporting. For the 36 patients with mucocutaneous infections and information available, 16 of 36 (44%) were deceased at the time of reporting. All 13 patients with disseminated or CNS infections were deceased at the time of reporting.

## 4. Discussion

Our systematic review of reports on dematiaceous fungal infections with individualized patient data in HSCT recipients and patients with predisposing hematological conditions identified 88 infections caused by over 25 genera. More than three-fourths of patients had leukemia, lymphoma, or underwent HSCT, and antifungal prophylaxis was used in 83% of the cases with information available. The most common syndromes were mucocutaneous infection (45%), pulmonary infection (14%), and sinusitis (13%). These represented breakthrough mold infections for 64% of patients on antifungal prophylaxis. For management, more than one antifungal agent was used in roughly half of infections, and 42% underwent surgical or procedural interventions. Two-thirds of the patients with information available were deceased at the time of report. Although much less common than aspergillosis and mucormycosis, the present review supports dematiaceous fungal infections as capable of causing significant morbidity in this patient population.

When comparing HSCT recipients with non-HSCT recipients, there was a larger proportion of pulmonary infections (35% vs. 2%) and a lower relative proportion of mucocutaneous infections (19% vs. 60%). The observed number of pulmonary infections in HSCT recipients may in part relate to sampling artifact, as one retrospective study on *Microascus* accounted for 82% (9/11) of cases [42]. The difference in the proportion of mucocutaneous infections may be partially explained by exposures to plant matter and/or relevant occupations or hobbies, as 93% of patients with these exposures were non-HSCT recipients. The male predominance in both groups is consistent with prior reports on dematiaceous fungal infections, and this may relate to gender differences in occupations [10,19]. Observed differences in clinical syndromes were likely impacted by net states of immunosuppression, as the immune system of HSCT recipients is markedly different from that of pre-transplant patients with hematological conditions. For example, risk factors for invasive fungal infections in HSCT recipients include GVHD or receipt of T cell active immunosuppression whereas clinical factors such as prolonged neutropenia or treatment-refractory disease heighten non-HSCT recipients’ risk profile [31]. Newer targeted therapies such as venetoclax or ibrutinib further modulate immune responses to fungi in patients with hematological conditions and may alter clinical manifestations of dematiaceous fungi [133]. Additionally, differences in the use of antifungal prophylaxis may have affected the observed species and their associated clinical syndromes. The inciting fungal species is important to consider, and certain observations may be explained by fungal isolates. For example, all *L. prolificans* infections occurred in non-HSCT recipients and raised the observed number of disseminated infections. The all-cause mortality observed in this review was likely to have been influenced by the prognoses associated with various hematological conditions and complications of HSCT.

Invasive fungal infections are known to disproportionately affect patients with hematological malignancies experiencing prolonged neutropenia and HSCT recipients requiring treatment of GVHD [134]. Antifungal prophylaxis is often recommended for intermediate to high-risk patients. The risk of fungal infection is impacted by the duration of neutropenia, exposure to cytotoxic chemotherapy, disease control of the underlying malignancy, increased immunosuppression for GVHD treatment and the use of antifungal prophylaxis. To ensure adequate serum levels of azole prophylaxis, therapeutic dose monitoring is available for isavuconazole, itraconazole, posaconazole, and voriconazole [134,135]. Breakthrough infections can occur despite primary antifungal prophylaxis, and these infections may be associated with resistant fungal species, suboptimal drug levels and impaired host immune responses including from prolonged neutropenia, mucositis, and central venous catheters [136]. For molds, specifically, breakthrough infections have been reported to range from 0 to 11% for posaconazole as primary prophylaxis and <1–8% for voriconazole as primary or secondary prophylaxis [137]. The true incidence of breakthrough mold infection in our dataset is unable to be determined due to incomplete reporting on antifungal prophylaxis and reporting bias associated with these cases, but the reported use of fluconazole in roughly one-fourth of cases (8/30; 27%) would not have offered coverage for dematiaceous molds. Based on the wide range of susceptibility profiles across dematiaceous mold species and individual isolates, it is challenging to offer uniformly effective azole prophylaxis, but isavuconazole, itraconazole, posaconazole, and voriconazole would be expected to have the most reliable activity [25,138].

Indeed, for purposes of prophylaxis or treatment, the susceptibility patterns of dematiaceous fungi lead to major challenges as there are no standardized clinical breakpoints or ECVs for most genera. To our knowledge, only two species of *Fonsecaea* have CLSI ECVs available [27,28]. The ECV aims to define a distribution of wild-type MICs for a fungal species so that phenotypic evidence for an acquired resistance mechanism can be inferred when comparing new isolates to the known MIC distribution. ECVs are not clinically used under normal circumstances as clinicians most often rely upon clinical breakpoints [139]. In contrast to ECVs, clinical breakpoints allow for isolates to be labeled as susceptible or not susceptible to a given antifungal agent, and the standardization of clinical breakpoints requires a robust review process encompassing ECV data, pharmacokinetic–pharmacodynamic data, and clinical data that attempts to discern the relation of MICs to clinical outcomes. No dematiaceous fungal species have clinical breakpoints endorsed by CLSI or EUCAST. Nonetheless, clinicians must treat patients with dematiaceous fungal infections, and MIC data offer insight into which agents have better potential for in vivo activity. In the absence of clinical breakpoints and ECVs, there are online repositories (e.g., https://mycology.adelaide.edu.au/fungal-descriptions-and-antifungal-susceptibility accessed 23 August 2026) of antifungal susceptibility data which provide MICs for uncommonly described species, and these data can be compared to a particular clinical isolate. Published MIC data on select species may suggest resistance to several classes of antifungal agents, and this can inform treatment strategies. For example, MIC data in this review identified *Microascus* and *L. prolificans* as having elevated MICs to several antifungal agents, and this has been previously observed [140,141,142]. The novel agent olorofilm, a dihydroorotate dehydrogenase inhibitor, has lower in vitro MICs for these isolates, so further data may support its role as an agent for infections due to *L. prolificans* and *Microascus* [142,143,144].

In contrast to our prior systematic review on dematiaceous fungal infections in solid organ transplant recipients (n = 201), the available literature had fewer dematiaceous fungal infections in HSCT recipients and patients with predisposing hematological conditions [13]. This was a counterintuitive finding based on the risk profile of patients with prolonged neutropenia, and a prior report on a prospective cohort of HSCT and solid organ transplant recipients reported similar numbers of phaeohyphomycosis cases (26 vs. 30) [121]. There are a few possible explanations for this observation. First, the widespread use of mold-active prophylaxis in the current patient population likely affects the incidence of disease as routine use of anti-mold prophylaxis is not recommended under most circumstances for solid organ transplant recipients other than lung transplant recipients [29]. Second, the diagnosis of invasive fungal disease often relies upon histopathology demonstrating host–pathogen interaction [41], and tissue-based diagnostics are occasionally deferred due to the perceived risk of complication in hematological patients with thrombocytopenia and/or coagulopathy. For example, bronchoscopy with or without biopsy is often viewed as higher risk for patients with severe thrombocytopenia [145]. This may lead to underdiagnosis and empiric therapy without mycological confirmation. Third, solid organ transplant recipients can survive for decades after transplantation [146], whereas the survival time for several hematological conditions, with the exception of HSCT recipients [147], is often measured in months to years. Longer survival times may translate to higher cumulative incidence of dematiaceous fungal infections as solid organ transplant recipients generally remain on indefinite immunosuppression for the duration of an allograft’s lifetime. Finally, publication bias may have impacted this observed discrepancy.

When comparing the current dataset’s clinical characteristics to our prior systematic review on solid organ transplant recipients, we note a higher proportion of pulmonary infections (14% vs. 5%) with a lower relative proportion of SSTI (45% vs. 73%) and CNS infections (5% vs. 11%) [13]. It is interesting to note that a prospective cohort study also observed a higher rate of pulmonary infection with a lower rate of SSTI in HSCT recipients with dematiaceous fungal infections relative to solid organ transplant recipients [121]. Sinusitis was also more commonly observed in the present cohort. These observations on the clinical behavior of dematiaceous fungi may in part relate to which arms of the immune system are altered. Neutrophils are integral to host defense against *Candida* and *Aspergillus*, and neutrophils can internalize and then destroy *Aspergillus* conidia or prevent the growth of hyphae [148]. For endemic mycoses and other fungi such as *Pneumocystis*, T cells and other immune effector cells are more important than neutrophils [149]. The net state of immunosuppression for HSCT recipients and patients with hematological malignancies is modulated by different mechanisms relative to solid organ transplant recipients, and this may impact tissue-invasive disease caused by dematiaceous fungi. For example, prolonged neutropenia related to hematological malignancy may allow for inhaled fungal conidia to more readily establish a foothold in sinus cavities or distal airways as the lower relative number of neutrophils leads to lower fungal clearance. This may in part account for a higher rate of sinopulmonary infections. In the current review, it is also important to recognize that one retrospective, single-center study on pulmonary *Microascus* infections disproportionately impacted the number of observed pulmonary infections [42], and further data are needed to confirm or refute the observation that HSCT recipients experience a higher number of pulmonary infections.

Despite the systematic approach, this review has several limitations. First, our review’s breadth is limited by the selective inclusion of reports with individualized patient data. We chose this approach to optimize the granularity of available data. Nonetheless, missing data and the level of data heterogeneity were marked, and these observations highlight the need for standardized case report formats that clearly delineate treatment and outcome. We did not perform a meta-analysis on this dataset as the proportion of missing data for key variables compromised the validity of this method, and data imputation may have led to spurious results. Second, we opted not to report mortality attributable to infection, as non-prospective reporting of cases in the available literature may have overestimated or underestimated these data. The definitive cause of mortality was infrequently reported, and the base rate of mortality for several hematological conditions is much higher than the general population. While all-cause mortality for disseminated and CNS infections was higher than that reported for mucocutaneous infections, this observation is subject to reporting bias. Third, the high proportion of missing data for follow-up duration and treatment duration limits the conclusions able to be drawn from this dataset. Prospective multinational data collection protocols are needed to adequately power an analysis that can better inform the most effective management strategies for these infections. Fourth, characterization of cases as proven, probable, or possible using 2020 criteria may have affected case classification for resource-limited settings or older reports without access to all contemporary diagnostics [41]. Finally, we were unable to retrieve three reports and excluded non-English publications, which accounted for 4% (33/769) of search results, so eligible cases may have been missed.

## 5. Conclusions

In this systematic review of published cases, dematiaceous fungi caused a range of clinical syndromes in patients with hematological malignancies and HSCT recipients. Breakthrough infections were common despite mold-active antifungal prophylaxis, highlighting the challenges in effectively preventing these infections. Antifungal susceptibility testing is needed to guide effective antifungal therapy; however, the absence of ECVs and clinical breakpoints limits its applicability. Surgical and procedural interventions are beneficial for tissue confirmation of phaeohyphomycosis and to lower the burden of infection in patients with altered immunity and neutropenia in whom antifungal therapy alone may be insufficient. Given the uncommon nature of dematiaceous fungal infections, prospective multicenter cohort studies are needed to better define recommended lengths of therapy, assess the relative effectiveness of antifungal combination therapy versus monotherapy, and strengthen the evidence base underpinning future consensus guidelines.

## Figures and Tables

**Table 1:** Demographics and clinical data for dematiaceous fungal infections.

	HSCT Recipient (n = 31)	Non-HSCT Recipient (n = 57)	Total (n = 88)
Median age in years (range)	42 (21–72)	62 (18–84) *	58 (18–84) *
Female (%)	15/31 (48%)	19/57 (33%)	34/88 (39%)
Neutropenia < 500/μL (%)	8/13 (62%)	9/17 (53%)	17/30 (57%)
Antifungal prophylaxis used (%)	19/20 (95%)	6/10 (60%)	25/30 (83%)
Mold-active prophylaxis used (%)	14/20 (70%)	2/10 (20%)	16/30 (53%)
Type of hematopoietic stem cell transplant			
Allogeneic (%)	18/31 (58%)	NA	NA
Autologous (%)	4/31 (13%)	NA	NA
Not reported (%)	9/31 (29%)	NA	NA
Median time post-HSCT in months (range)	23.5 (0.2–132) †	NA	NA
Relapsed pre-transplant disease (%)	7/31 (23%)	NA	NA
Graft-versus-host disease requiring treatment (%)	10/13 (77%)	NA	NA
Hematological condition			
Aplastic anemia (%)	NA	4/57 (7%)	NA
Leukemia (%)	NA	22/57 (39%)	NA
Lymphoma (%)	NA	18/57 (32%)	NA
**Myelodysplastic syndrome (%)**	NA	4/57 (7%)	NA
Other (%)	NA	9/57 (16%)	NA
EORTC/MSGERC 2020 classification [41]			
Proven (%)	19/31 (61%)	50/57 (88%)	69/88 (78%)
Probable (%)	1/31 (3%)	0	1/88 (1%)
Possible (%)	11/31 (35%)	7/57 (12%)	18/88 (20%)
Syndrome			
Mucocutaneous (%)	6/31 (19%)	34/57 (60%)	40/88 (45%)
Pulmonary (%)	11/31 (35%)	1/57 (2%)	12/88 (14%)
Disseminated (%)	2/31 (6%)	7/57 (12%)	9/88 (10%)
Sinusitis (%)	5/31 (16%)	6/57 (11%)	11/88 (13%)
Fungemia (%)	4/31 (13%)	2/57 (4%)	6/88 (7%)
Miscellaneous (%)	2/31 (6%)	4/57 (7%)	6/88 (7%)
Central nervous system (%)	1/31 (3%)	3/57 (5%)	4/88 (5%)
Systemic antifungal therapy administered (%)	31/31 (100%)	50/57 (88%)	81/88 (92%)
2 or more antifungal agents used (%)	19/30 (63%)	28/57 (49%)	47/87 (54%)
Surgical and/or procedural intervention (%)	7/31 (23%)	30/57 (53%)	37/88 (42%)
Median duration of follow-up in months (range)	1.1 (0.4–5.7) ‡	4 (0.4–44.4) §	3 (0.4–44.4) ¶
Death at time of report (%)	18/24 (75%)	29/51 (57%)	47/75 (63%)

* n = 4 missing data. † n = 7 missing data. ‡ n = 21 missing data. § n = 32 missing data. ¶ n = 53 missing data. Table denominators denote the number of cases for which information was available. The “other” group includes patients with chronic lymphocytic leukemia (n = 4), multiple myeloma (n = 2), myeloproliferative neoplasms (n = 2), and overlap syndromes (n = 1). Definitions: HSCT, hematopoietic stem cell transplantation; EORTC/MSGERC, European Organization for Research and Treatment of Cancer/Mycoses Study Group Education and Research Consortium; NA, not applicable.

**Table 2:** Antifungal susceptibility testing for 45 unique isolates.

Isolate	Standards Organization	Method	Fluconazole MIC (μg/mL)	Itraconazole MIC (μg/mL)	Voriconazole MIC (μg/mL)	Posaconazole MIC (μg/mL)	Isavuconazole MIC (μg/mL)	Anidulafungin MIC (μg/mL)	Caspofungin MIC (μg/mL)	Micafungin MIC (μg/mL)	Amphotericin B MIC (μg/mL)	Flucytosine MIC (μg/mL)	Terbinafine MIC (μg/mL)
***Alternaria*** ***alternata***	not reported	broth dilution		0.5	4	0.25					2		2
***Alternaria rosae***	not reported	not reported		1	2	0.25					0.5		
***Alternaria* spp.**	CLSI	broth microdilution		1	4	0.5	≥8	0.12	0.12	≤0.06	2		
***Alternaria*** ***tenuissima***	not reported	not reported									0.2	>100	
***Chaetomium*** ***globosum***	CLSI	microdilution	32	0.25							0.25	>128	
***Chaetomium*** ***globosum***	CLSI	microdilution	32	0.5							2	>128	
***Cladosporium cladosporioides***	EUCAST	ETEST		0.75	1			3		4			
***Colletotrichum coccodes***	CLSI	not reported	>64	2							0.5	32	
***Colletotrichum coccodes***	CLSI	not reported		0.25							0.06	8	
***Colletotrichum gloeosporioides***	CLSI	not reported	>64	0.5							0.5	32	
***Colletotrichum siamense***	CLSI	broth dilution			2	1	0.5				0.5		
***Curvularia* spp.**	CLSI	broth microdilution		2	4	0.5	≥8	1	2	0.5	2		
***Exophiala*** ***dermatitidis***	not reported	ASTY	8	0.25	0.06				4	8	0.25	2	
***Exophiala*** ***dermatitidis***	not reported	ASTY	8	0.125	0.06				16	16	0.25	>64	
***Exophiala*** ***dermatitidis***	CLSI	not reported	16							>16	0.5		
***Exophiala*** ***dermatitidis***	not reported	not reported	8	0.25	0.125	0.125		8	8	8	1	>64	0.06
***Exophiala*** ***dermatitidis***	not reported	not reported	>64	2	1					>16	2	4	
***Exophiala*** ***dermatitidis***	not reported	ETEST	16	0.25							0.032		
***Exophiala*** ***salmonis***	CLSI	microdilution		0.125	0.25						1		
***Exophiala*** ***spinifera***	not reported	not reported		0.03	0.25	<0.015					0.125		
***Exophiala*** ***xenobiotica***	CLSI	not reported	16	≤0.015						16	0.5	8	
***Exophiala*** ***xenobiotica***	CLSI	not reported	64	0.1						>16	2	32	
***Exserohilum rostratum***	not reported	not reported		0.125	1	0.06					0.5		
***Lomentospora prolificans***	not reported	not reported	>64	>8							4	>64	
***Lomentospora prolificans***	CLSI	micromethod		>8	>8	>8			8		8		>16
***Lomentospora prolificans***	CLSI	not reported	>64	>8	1					1	4	>64	
***Lomentospora prolificans***	CLSI	broth dilution	>100	>2							>8	>64	
***Medicopsis romeroi***	not reported	ETEST	>256	3	0.008	0.064		0.5	6		8		
***Microascus* spp.**	CLSI	broth microdilution	>64						0.5		>64	>64	0.125
***Microascus* spp.**	CLSI	broth microdilution	>64						8		>64	>64	0.125
***Microascus* spp.**	CLSI	broth microdilution	>64						8		>64	>64	0.125
***Microascus* spp.**	CLSI	broth microdilution	>64						8		>64	>64	0.125
***Microascus* spp.**	CLSI	broth microdilution	>64						8		>64	>64	0.125
***Microascus* spp.**	CLSI	broth microdilution	>64						8		>64	>64	0.125
***Microascus* spp.**	CLSI	broth microdilution	>64						>16		>64	>64	0.125
***Paraconiothyrium fuckelii***	not reported	not reported									0.1	12.5	
***Phaeoacremonium*** ***parasiticum***	CLSI	broth microdilution		1	0.125	0.25					0.5		
***Phialophora richardsiae***	not reported	not reported									1.56		
***Phialophora*** ***verrucosa***	CLSI	macrodilution	10	0.78							0.1	>20	
***Pleurostoma richardsiae***	not reported	broth microdilution	128	1	0.5	1		8		8	4	>64	
***Pleurostoma richardsiae***	not reported	not reported	>64	>8	>8					0.5	>16	>64	
***Thermothelomyces thermophilus***	EUCAST	not reported		0.125	0.125	0.125			4		2		
***Triadelphia*** ***pulvinata***	CLSI	not reported		2	1	1			0.5		0.25		
***Verruconis*** ***gallopava***	not reported	not reported	64	0.5	1						2	16	
***Verruconis* spp.**	CLSI	broth dilution		0.25		0.125	>16	≤0.015					

For the fungal isolates in this table, there are no ECVs or clinical breakpoints available for MIC interpretation. Antimicrobial susceptibility testing methods are listed as reported. The standards organizations formerly referred to as National Committee for Clinical Laboratory Standards is referred to as CLSI in this table as the organization changed its name in 2005. The ASTY panel (Kyokuto Pharmaceutical Industrial Co., Tokyo, Japan) is a colorimetric microdilution panel [131], and the ETEST (bioMérieux, Marcy-l’Étoile, France) refers to the Epsilometer test that uses a test strip with a gradient of the antimicrobial [132].

## Data Availability

Dataset available on request from the authors.

## Abbreviations

The following abbreviations are used in this manuscript:

BMD	Broth microdilution
CLSI	Clinical & Laboratory Standards Institute
CNS	Central nervous system
ECV	Epidemiological cut-off value
EORTC	European Organization for Research and Treatment of Cancer
EUCAST	European Committee of Antimicrobial Susceptibility Testing
GVHD	Graft-versus-host disease
HSCT	Hematopoietic stem cell transplantation
MALDI-TOF MS	Matrix-assisted laser desorption/ionization time of flight mass spectrometry
MIC	Minimum inhibitory concentration
MSGERC	European Organization for Research and Treatment of Cancer
SSTI	Skin and soft tissue infection

## Appendix A

The following materials relate to the 2020 Preferred Reporting Items for Systematic Reviews and Meta-Analyses (PRISMA). Table A1 presents our review’s PRISMA checklist for applicable sections of this manuscript, and Table A2 presents individualized data on the 74 reports included in this systematic review.

**Table A1:** 2020 PRISMA Checklist.

Section and Topic	Item #	Checklist Item	Location Where Item Is Reported
**TITLE**			
Title	1	Identify the report as a systematic review.	Title
**ABSTRACT**			
Abstract	2	See the PRISMA 2020 for Abstracts checklist.	Abstract
**INTRODUCTION**			
Rationale	3	Describe the rationale for the review in the context of existing knowledge.	Introduction
Objectives	4	Provide an explicit statement of the objective(s) or question(s) the review addresses.	Introduction
**METHODS**			
Eligibility criteria	5	Specify the inclusion and exclusion criteria for the review and how studies were grouped for the syntheses.	Methods Section 2.1
Information sources	6	Specify all databases, registers, websites, organizations, reference lists and other sources searched or consulted to identify studies. Specify the date when each source was last searched or consulted.	Methods Section 2.1
Search strategy	7	Present the full search strategies for all databases, registers and websites, including any filters and limits used.	Methods Section 2.1
Selection process	8	Specify the methods used to decide whether a study met the inclusion criteria of the review, including how many reviewers screened each record and each report retrieved, whether they worked independently, and if applicable, details of automation tools used in the process.	Methods Section 2.1 and Section 2.2
Data collection process	9	Specify the methods used to collect data from reports, including how many reviewers collected data from each report, whether they worked independently, any processes for obtaining or confirming data from study investigators, and if applicable, details of automation tools used in the process.	Methods Section 2.2
Data items	10a	List and define all outcomes for which data were sought. Specify whether all results that were compatible with each outcome domain in each study were sought (e.g., for all measures, time points, analyses), and if not, the methods used to decide which results to collect.	Methods Section 2.2
	10b	List and define all other variables for which data were sought (e.g., participant and intervention characteristics, funding sources). Describe any assumptions made about any missing or unclear information.	Methods Section 2.1 and Section 2.2
Study risk of bias assessment	11	Specify the methods used to assess risk of bias in the included studies, including details of the tool(s) used, how many reviewers assessed each study and whether they worked independently, and, if applicable, details of automation tools used in the process.	Methods Section 2.1
Effect measures	12	Specify for each outcome the effect measure(s) (e.g., risk ratio, mean difference) used in the synthesis or presentation of results.	Not applicable
Synthesis methods	13a	Describe the processes used to decide which studies were eligible for each synthesis (e.g., tabulating the study intervention characteristics and comparing against the planned groups for each synthesis (item #5)).	Methods Section 2.3
	13b	Describe any methods required to prepare the data for presentation or synthesis, such as handling of missing summary statistics, or data conversions.	Methods Section 2.3
	13c	Describe any methods used to tabulate or visually display results of individual studies and syntheses.	Methods Section 2.3
	13d	Describe any methods used to synthesize results and provide a rationale for the choice(s). If meta-analysis was performed, describe the model(s), method(s) to identify the presence and extent of statistical heterogeneity, and software package(s) used.	Methods Section 2.3
	13e	Describe any methods used to explore possible causes of heterogeneity among study results (e.g., subgroup analysis, meta-regression).	Not applicable
	13f	Describe any sensitivity analyses conducted to assess robustness of the synthesized results.	Not applicable
Reporting bias assessment	14	Describe any methods used to assess risk of bias due to missing results in a synthesis (arising from reporting biases).	Methods Section 2.1
Certainty assessment	15	Describe any methods used to assess certainty (or confidence) in the body of evidence for an outcome.	Methods Section 2.1
**RESULTS**			
Study selection	16a	Describe the results of the search and selection process, from the number of records identified in the search to the number of studies included in the review, ideally using a flow diagram.	Results Section 3.1, Figure 1
	16b	Cite studies that might appear to meet the inclusion criteria, but which were excluded, and explain why they were excluded.	Results Section 3.1
Study characteristics	17	Cite each included study and present its characteristics.	Results Section 3.1, Table 1
Risk of bias in studies	18	Present assessments of risk of bias for each included study.	Results Section 3.1, Table A2
Results of individual studies	19	For all outcomes, present, for each study: (a) summary statistics for each group (where appropriate) and (b) an effect estimate and its precision (e.g., confidence/credible interval), ideally using structured tables or plots.	Results Section 3.1, Table 1 and Table A2
Results of syntheses	20a	For each synthesis, briefly summarize the characteristics and risk of bias among contributing studies.	Results Section 3.1
	20b	Present results of all statistical syntheses conducted. If meta-analysis was done, present for each the summary estimate and its precision (e.g., confidence/credible interval) and measures of statistical heterogeneity. If comparing groups, describe the direction of the effect.	Results Section 3.1
	20c	Present results of all investigations of possible causes of heterogeneity among study results.	Not applicable
	20d	Present results of all sensitivity analyses conducted to assess the robustness of the synthesized results.	Not applicable
Reporting biases	21	Present assessments of risk of bias due to missing results (arising from reporting biases) for each synthesis assessed.	Not applicable
Certainty of evidence	22	Present assessments of certainty (or confidence) in the body of evidence for each outcome assessed.	Results Section 3.1
**DISCUSSION**			
Discussion	23a	Provide a general interpretation of the results in the context of other evidence.	Discussion
	23b	Discuss any limitations of the evidence included in the review.	Discussion
	23c	Discuss any limitations of the review processes used.	Discussion
	23d	Discuss implications of the results for practice, policy, and future research.	Discussion
**OTHER INFORMATION**			
Registration and protocol	24a	Provide registration information for the review, including register name and registration number, or state that the review was not registered.	Methods Section 2.1
	24b	Indicate where the review protocol can be accessed, or state that a protocol was not prepared.	Methods Section 2.1
	24c	Describe and explain any amendments to information provided at registration or in the protocol.	Methods Section 2.1
Support	25	Describe sources of financial or non-financial support for the review, and the role of the funders or sponsors in the review.	Funding statement
Competing interests	26	Declare any competing interests of review authors.	Conflicts of interest statement
Availability of data, code and other materials	27	Report which of the following are publicly available and where they can be found: template data collection forms; data extracted from included studies; data used for all analyses; analytic code; any other materials used in the review.	Data availability statement

**Table A2:** Included reports on dematiaceous fungal infections.

PMID	Authors’ Country of Origin	Study Design	Was the Exposure Adequately Ascertained? (Yes 1, No 0)	Was the Outcome Adequately Ascertained? (Yes 1, No 0)	Is the Case(s) Described with Sufficient Details to Allow Other Investigators to Replicate the Research or to Allow Practitioners to Make Inferences Related to Their Own Practice? (Yes 1, No 0)	Was Follow-Up Long Enough for Outcomes to Occur? (Yes 1, No 0)	Modified Bias Assessment (Max Score 4)	GRADE Guideline Quality	Number of Cases Included	Age (Years)	Sex (1 Female, 0 Male)	Stem Cell Transplant Recipient (Yes 1, No 0)	Clinical Syndrome	Fungal Isolate(s)	Outcome at Time of Report (Alive 1, Dead 2, Not Reported 3)
41624956	Multiple	Case report	0	1	1	1	3	very low	1	72	1	1	Pulmonary	*Verruconis*	2
41718310	Bulgaria	Case report	0	1	1	1	3	very low	1	61	0	0	Fungemia	*Cladosporium cladosporioides*	1
40071988	France	Case report	0	0	1	0	1	very low	1	72	0	0	Mucocutaneous	*Alternaria alternata* species complex	1
38991295	USA	Case report	0	1	1	1	3	very low	1	35	0	1	Sinusitis	*Alternaria* & *Curvularia*	2
37845401	USA	Case report	0	0	1	0	1	very low	1	39	0	1	Sinusitis	*Bipolaris*	1
38182155	USA	Case report	1	0	1	0	2	very low	1	57	0	0	Sinusitis	*Exserohilum rostratum*	1
34765385	Australia	Case report	1	0	1	0	2	very low	1	82	0	0	Mucocutaneous	*Pleurostoma richardsiae*	3
33768850	Japan	Case report	0	1	1	1	3	very low	1	44	0	0	Disseminated	*Lomentospora prolificans*	2
33034837	USA	Case report	1	0	1	0	2	very low	1	72	0	0	Mucocutaneous	*Microsphaeropsis arundinis*	1
31357231	Multiple	Case report	1	1	1	1	4	very low	1	66	1	1	Mucocutaneous	*Colletotrichum siamense*	2
30986396	USA	Case report	1	1	1	1	4	very low	1	77	0	0	Mucocutaneous	*Exophiala jeanselmei*	1
30996161	Japan	Case report	0	0	1	0	1	very low	1	69	1	0	Fungemia	*Exophiala dermatitidis*	1
30679025	Japan	Case report	1	1	1	1	4	very low	1	29	0	1	Disseminated	*Exophiala dermatitidis*	2
30449799	Japan	Case report	1	1	1	1	4	very low	1	21	1	1	Disseminated	*Exophiala dermatitidis*	2
29215152	Japan	Case report	1	1	1	1	4	very low	1	84	0	0	Mucocutaneous	*Veronaea botryosa*	1
29359870	Japan	Case report	0	1	1	1	3	very low	1	45	0	1	Fungemia	*Exophiala dermatitidis*	2
29119669	Japan	Case report	0	0	1	0	1	very low	1	60	0	1	Sinusitis	*Exserohilum rostratum*	1
28295973	USA	Case report	1	1	1	1	4	very low	1	66	0	1	Mucocutaneous	*Alternaria rosae*	2
27456474	France	Case report	1	1	1	1	4	very low	1	76	0	0	Mucocutaneous	*Exophiala spinifera*	1
26531188	USA	Case report	1	0	1	0	2	very low	1	not reported	1	0	Mucocutaneous	*Curvularia*	1
24890324	USA	Case report	1	1	1	1	4	very low	1	57	0	1	Fungemia	*Exophiala dermatitidis*	2
23863568	Multiple	Case report	1	1	1	1	4	very low	1	58	1	1	Fungemia	*Triadelphia pulvinata*	2
24371750	Japan	Case report	1	0	1	0	2	very low	1	65	0	0	Pulmonary	*Exophiala dermatitidis*	1
21852529	China	Case report	1	1	1	1	4	very low	1	38	0	0	Central nervous system	*Cladophialophora bantiana*	2
21455664	Spain	Case report	0	1	1	1	3	very low	1	29	1	0	Disseminated	*Lomentospora prolificans*	2
21429692	France	Case report	0	1	1	1	3	very low	1	65	0	0	Disseminated	*Thermothelomyces thermophilus*	2
20594890	USA	Case report	0	1	1	1	3	very low	1	32	1	0	Sinusitis	*Exserohilum*	2
20398199	Japan	Case report	0	1	1	1	3	very low	1	58	1	0	Disseminated	*Lomentospora prolificans*	2
19107636	Japan	Case report	1	1	1	1	4	very low	1	77	1	0	Mucocutaneous	*Exophiala xenobiotica*	2
16757622	USA	Case report	0	1	1	1	3	very low	1	31	1	0	Mucocutaneous	*Phaeoacremonium parasiticum*	2
16220103	USA	Case report	0	1	1	1	3	very low	1	18	1	0	Sinusitis	*Exserohilum rostratum*	2
16157992	Japan	Case report	1	1	1	1	4	very low	1	not reported	1	0	Disseminated	*Verruconis gallopava*	2
14682456	Multiple	Case report	1	1	1	1	4	very low	1	34	0	1	Lymphadenitis	*Chaetomium globosum*	2
12353348	Taiwan	Case report	1	1	1	1	4	very low	1	62	1	0	Lymphadenitis	*Exophiala dermatitidis*	1
11270415	Lebanon	Case report	0	1	1	1	3	very low	1	71	0	0	Central nervous system	*Rhinocladiella mackenziei*	2
10321944	Thailand	Retrospective	1	1	1	1	4	very low	1	52	0	0	Mucocutaneous	*Cladophialophora carrionii*	1
9384484	USA	Case report	1	1	1	1	4	very low	1	42	1	1	Tracheitis	*Phialophora verrucosa*	2
9145005	Germany	Case report	1	1	0	1	3	very low	1	60	0	0	Disseminated	*Lomentospora prolificans*	2
1510217	USA	Case report	0	1	1	1	3	very low	1	71	0	0	Mucocutaneous	*Phaeoacremonium parasiticum* & *Fonsecaea pedrosoi*	3
2802647	USA	Case report	1	0	1	0	2	very low	1	35	1	0	Mucocutaneous	*Curvularia spicifera*	1
3170810	Japan	Case report	1	1	1	1	4	very low	1	30	0	0	Mucocutaneous	*Pleurostoma richardsiae*	2
3480895	USA	Case report	1	1	1	1	4	very low	1	59	1	0	Liver nodules	*Paraconiothyrium fuckelii*	2
3540674	Italy	Case report	0	1	1	1	3	very low	2	54	1	0	Mucocutaneous	*Alternaria tenuissima*	2
										52	0	0	Mucocutaneous	*Alternaria tenuissima*	1
3461148	Japan	Case report	1	1	1	1	4	very low	1	58	1	0	Mucocutaneous	*Verruconis gallopava*	2
3860191	USA	Case report	0	1	1	1	3	very low	1	54	1	0	Mucocutaneous	*Myrmecridium schulzeri*	2
41113642	China	Retrospective	0	0	1	0	1	very low	9	22	1	1	Pulmonary	*Microascus*	3
										33	1	1	Pulmonary	*Microascus*	2
										58	1	1	Pulmonary	*Microascus*	3
										36	1	1	Pulmonary	*Microascus*	3
										51	0	1	Pulmonary	*Microascus*	3
										39	1	1	Pulmonary	*Microascus*	3
										40	0	1	Pulmonary	*Microascus*	2
										34	1	1	Pulmonary	*Microascus*	3
										57	0	1	Pulmonary	*Microascus*	3
40252587	India	Case report	1	0	1	0	2	very low	1	38	0	1	Mucocutaneous	*Parathyridaria percutanea*	1
41285445	USA	Case report	1	1	1	1	4	very low	1	76	0	0	Mucocutaneous	*Exophiala bergeri*	1
39660859	USA	Case report	1	0	1	0	2	very low	1	60	0	0	Mucocutaneous	*Exophiala jeanselmei*	1
38272660	USA	Case series	0	0	1	0	1	very low	2	not reported	0	0	Mucocutaneous	*Alternaria*	2
										not reported	0	0	Lymphadenitis	*Aureobasidium pullulans*	2
37661451	Japan	Case report	0	1	1	1	3	very low	1	71	0	1	Pulmonary	*Verruconis gallopava*	2
37381098	Spain	Retrospective	1	0	1	0	2	very low	1	70	0	0	Mucocutaneous	*Alternaria infectoria*	1
35600570	USA	Case report	1	0	1	0	2	very low	1	71	0	0	Mucocutaneous	*Exophiala*	3
No PMID—ISSN 10569103	USA	Case report	0	0	1	0	1	very low	2	33	0	0	Sinusitis	*Curvularia*	2
										58	1	1	Sinusitis	*Curvularia lunata*	1
32637729	USA	Case report	0	1	1	1	3	very low	1	67	0	0	Endophthalmitis	*Exophiala dermatitidis*	2
No PMID—ISSN 23710888	Canada	Case report	0	1	1	1	3	very low	1	22	1	1	Sinusitis	*Phoma*	2
28186363	Japan	Case report	0	1	1	1	3	very low	1	78	0	0	Mucocutaneous	*Pleurostoma richardsiae*	2
25538223	USA	Case report	0	0	1	0	1	very low	1	75	1	0	Sinusitis	*Curvularia*	3
24270260	Brazil	Case report	1	1	1	1	4	very low	1	66	0	0	Mucocutaneous	*Aureobasidium pullulans*	2
19836185	USA	Case report	0	0	1	0	1	very low	2	50	0	0	Mucocutaneous	*Curvularia*	3
										75	0	0	Sinusitis	*Exserohilum*	3
19261021	Taiwan	Case report	1	1	1	1	4	very low	1	55	0	0	Mucocutaneous	*Exserohilum rostratum*	2
17076729	Greece	Case report	1	1	1	1	4	very low	1	81	0	0	Mucocutaneous	*Exophiala jeanselmei*	1
22404860	France	Case series	0	1	1	1	3	very low	1	77	0	0	Mucocutaneous	*Alternaria infectoria* group	2
21299373	USA	Case report	1	1	1	1	4	very low	1	58	0	0	Mucocutaneous	*Alternaria alternata*	2
21349989	Kuwait	Case report	1	0	1	0	2	very low	1	47	1	0	Mucocutaneous	*Medicopsis romeroi*	1
17034552	UK	Case report	1	0	1	0	2	very low	1	64	0	0	Mucocutaneous	*Exophiala salmonis*	1
11423835	USA	Case series	1	0	1	0	2	very low	3	34	0	0	Mucocutaneous	*Colletotrichum gloeosporioides*	1
										47	0	1	Mucocutaneous	*Colletotrichum coccodes*	1
										58	0	1	Mucocutaneous	*Colletotrichum coccodes*	2
11452326	Brazil	Case report	0	1	1	1	3	very low	1	30	0	1	Mucocutaneous	*Thyridium curvatum*	1
8727934	USA	Case report	0	1	1	1	3	very low	1	31	1	1	Central nervous system	*Cladophialophora bantiana*	2
No PMID—ISSN 18147453	China	Case report	1	1	1	1	4	very low	1	72	1	0	Mucocutaneous	*Cladophialophora* spp. & *Exophiala jeanselmei*	1
22145276	Belgium	Case report	0	1	1	1	3	very low	1	54	1	1	Fungemia	*Thyridium curvatum*	2
No PMID—ISSN 12034754	USA	Case report	1	1	1	1	4	very low	1	72	0	0	Mucocutaneous	*Exophiala jeanselmei*	2
4739938	USA	Case report	0	1	0	1	2	very low	1	31	1	0	Central nervous system	*Curvularia hawaiiensis*	2
2360556	USA	Case report	1	1	0	1	3	very low	1	62	0	0	Disseminated	*Verruconis gallopava*	2

Articles for which PubMed identification (PMID) numbers were unavailable have a unique International Standard Serial Number (ISSN) recorded.
